# Correlation between serum prolactin and the systemic immune-inflammation index in diabetic kidney disease: a cross-sectional study

**DOI:** 10.3389/fendo.2026.1772810

**Published:** 2026-03-02

**Authors:** Yinhui Li, Zhonghao Qin, Tian Si, Shijie Wu, Haichao Wang, Hanjie Yu, Kaimin Wang, Lei Yang, Ai Peng, Minghao Kong, Ling Wang

**Affiliations:** 1 Center for Nephrology and Clinical Metabolomics and Division of Nephrology, Shanghai Tenth People’s Hospital, Tongji University School of Medicine, Shanghai, China; 2 Department of Laboratory, Shanghai Tenth People’s Hospital, Tongji University School of Medicine, Shanghai, China; 3 Department of Rheumatology, The Affiliated Hospital of Guizhou Medical University, Guiyang, Guizhou, China

**Keywords:** cross-sectional investigation, diabetic kidney disease, micro-inflammation, prolactin, systemic immune-inflammation index

## Abstract

**Objective:**

Prolactin (PRL) is increasingly recognized as a pleiotropic hormone with potent immunoregulatory properties; however, its involvement in systemic inflammation among diabetic kidney disease (DKD) patients has not been defined. This study aimed to investigate the potential association between serum PRL levels and micro-inflammation in patients with DKD.

**Methods:**

In this cross-sectional investigation, 994 patients with type 2 diabetes mellitus (T2DM)-associated DKD were enrolled. Multivariable linear mixed-effects models were used to quantify the relationship between serum PRL and the systemic immune-inflammation index (SII) and other clinical parameters. Restricted cubic spline (RCS) analyses were fitted to test for non-linearity and stratified and sensitivity analyses were performed to assess robustness.

**Results:**

The median serum PRL level was 344.40 mIU/L (interquartile range: 258.80–463.10). After multivariable adjustment, serum PRL were positively associated with white blood cell count, neutrophil count, serum ferritin, serum phosphorus and intact parathyroid hormone (iPTH) levels, and inversely associated with serum albumin. For every 100 mIU/L increase in serum PRL, the SII increased by an average of 7.10 units (95% CI: 3.13 to 11.07; *p* = 5.12 × 10^-4^). Stratified and sensitivity analyses confirmed the robustness of this association. RCS analysis revealed a significant nonlinear relationship between serum PRL and SII (*p* for nonlinearity = 0.002), with an inflection point at 282.85 mIU/L. PRL levels above this inflection point showed a significant positive association with SII, whereas levels below it showed a negative association.

**Conclusion:**

In patients with DKD, serum PRL exhibits an independent, nonlinear association with SII, characterized by a threshold around 282.85 mIU/L. This finding suggests that PRL may be linked to the dysregulated immune-inflammation axis in DKD, warranting further mechanistic and longitudinal investigation.

## Introduction

Diabetic kidney disease (DKD) is one of the most common and serious microvascular complications of diabetes, with a steadily increasing global prevalence. It affects approximately 40% of individuals with diabetes and has become the leading cause of chronic kidney disease (CKD) and end-stage renal disease (ESRD), accounting for 30% to 50% of all cases (1). More concerningly, data from the Global Burden of Disease study indicate that DKD is responsible for 30.7% of all CKD-related disability-adjusted life years (DALYs), ranking first among etiologies, with type 2 diabetes mellitus (T2DM) driving 73.6% of this burden (2, 3). DKD not only imposes a substantial burden on patients but also poses a major challenge to global public health systems. Therefore, elucidating the pathogenesis of DKD and identifying novel therapeutic targets are of great practical significance.

Beyond traditional metabolic and hemodynamic abnormalities, chronic low-grade systemic inflammation has recently been recognized as a key driver in the onset and progression of DKD (4). This “micro-inflammation” state is not caused by acute infection but is rather triggered by multiple factors, including metabolic disturbances, hemodynamic stress, and oxidative stress. It involves intricate interactions between resident renal and immune cells, forming a persistent yet subtle inflammatory network, establishing a persistent low-grade inflammatory milieu that promotes glomerulosclerosis and tubulointerstitial fibrosis, ultimately resulting in progressive renal function decline (4, 5). Renal biopsy samples from DKD patients frequently demonstrate infiltration of immune cells, primarily macrophages, along with persistently elevated expression of pro-inflammatory factors such as interleukin-6 (IL-6), monocyte chemoattractant protein-1 (MCP-1), and tumor necrosis factor-α (TNF-α) (5, 6). Notably, contemporary DKD management has entered an era focused on organ protection, including renin–angiotensin system (RAS) inhibitors, sodium-glucose cotransporter 2 (SGLT2) inhibitors, glucagon-like peptide-1 (GLP-1) receptor agonists, and non-steroidal mineralocorticoid receptor antagonists (MRAs) (7). The renoprotective effects of these agents are closely linked to their anti-inflammatory and immunomodulatory properties (8–10). For example, the non-steroidal mineralocorticoid receptor antagonist finerenone confers renal protection in part through inhibition of the central inflammatory NF-κB pathway (7). Collectively, effective identification and quantification of this micro-inflammation hold significant clinical importance for risk stratification and personalized targeted intervention in DKD.

Conventional inflammatory biomarkers, such as C-reactive protein (CRP), primarily reflect acute-phase responses and may be insufficiently sensitive to the chronic, low-grade, cell-mediated inflammatory state characteristic of DKD. The systemic immune-inflammation index (SII), which integrates neutrophil, lymphocyte, and platelet counts, provides a composite measure that more comprehensively captures the dynamic balance between pro-inflammatory activation, immune regulation, and thrombo-inflammatory interactions (11, 12). The prognostic value of SII has been validated in cardiovascular disease, autoimmune disorders, and various chronic inflammatory conditions (13–18), and recent studies have further demonstrated that elevated SII independently predicts more rapid estimated glomerular filtration rate (eGFR) decline and higher cardiovascular risk in patients with DKD (19–21). Accordingly, SII represents a practical and readily accessible surrogate marker for assessing systemic chronic micro-inflammation in this population.

Prolactin (PRL) is conventionally viewed as an anterior pituitary hormone, yet it also operates as a pleiotropic cytokine that orchestrates neuroendocrine signaling and both innate and adaptive immunity (22, 23). Hyperprolactinemia amplifies T- and B-cell activation and stimulates the release of TNF-α, interleukin-1β (IL-1β), and IL-6, thereby propagating organ-specific autoimmune injury (23). Clinically, elevated PRL concentrations are documented in 30-65% of patients with CKD and independently predict cardiovascular events and all-cause mortality (24, 25). Given that the progression of DKD is closely linked to the degree of renal inflammatory cell infiltration (1, 5), and that PRL receptor expression is significantly upregulated in the kidneys of diabetic rodents (26, 27), PRL emerges as a biologically plausible, yet under-recognized, contributor to the micro-inflammation that characterizes DKD. Importantly, rather than serving merely as a direct surrogate marker of inflammation, PRL may act as an upstream endocrine modulator that shapes systemic immune-inflammatory activity. We therefore undertook the present cross-sectional analysis to quantify the relationship between circulating PRL and SII in patients with established DKD.

## Methods

### Study design and ethical approval

This was a single-center, retrospective, cross-sectional study. This study was approved by the Ethics Committee of Shanghai Tenth People’s Hospital, Tongji University School of Medicine (Approval No. SHSY-IEC-5.0/23K42/P01) and conducted in accordance with the principles of the Declaration of Helsinki. The ethics committee waived the requirement for individual informed consent on the grounds that the study was retrospective and non-interventional, posed no unnecessary risk, and utilized fully anonymized patient data.

### Study population

**Figure 1:**
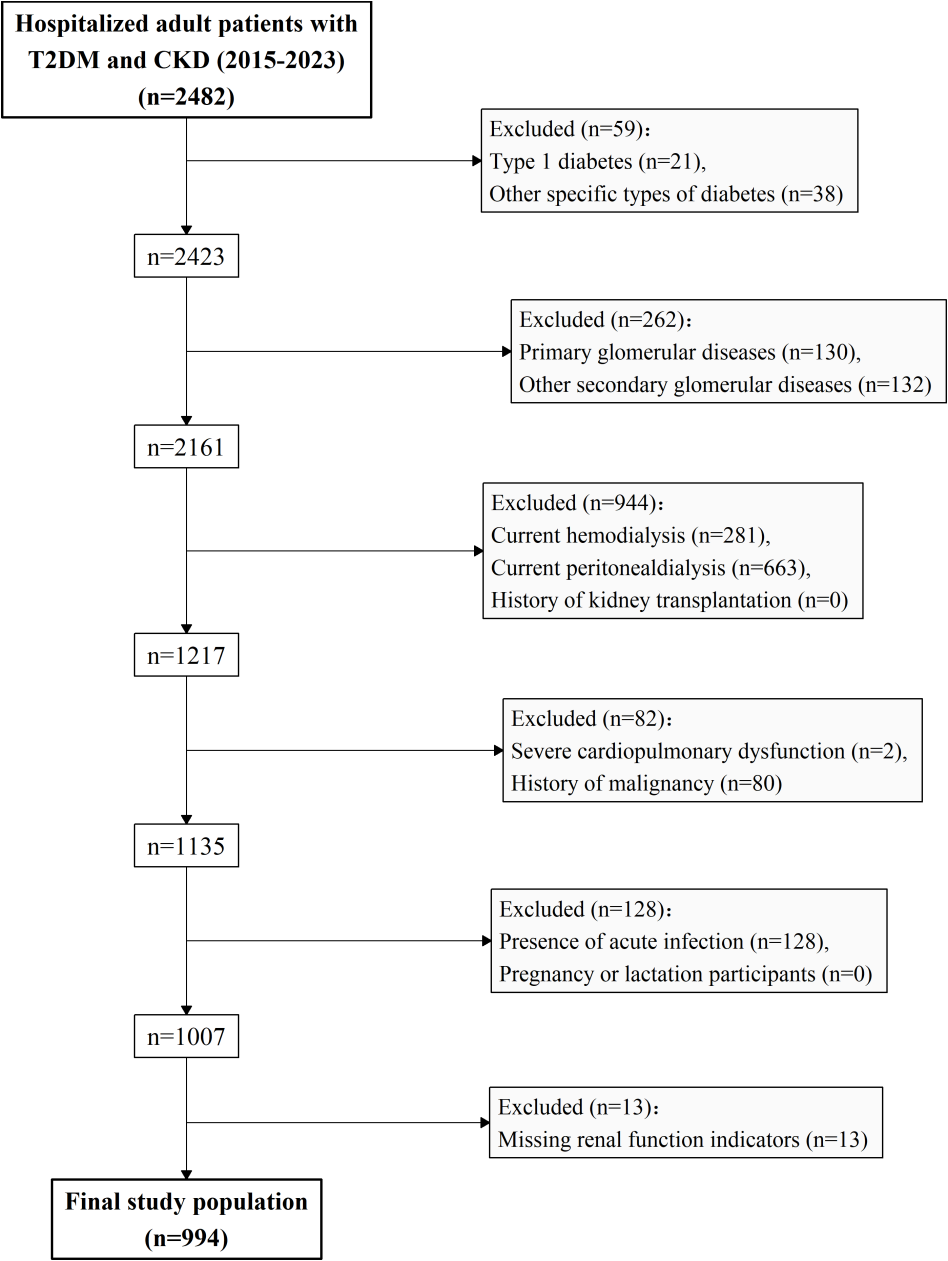
Flowchart of study population selection.

Adult patients hospitalized in the Division of Nephrology at Shanghai Tenth People’s Hospital between January 2015 and December 2023 who met the diagnostic criteria for CKD and T2DM were eligible for inclusion. CKD was defined according to the Kidney Disease: Improving Global Outcomes (KDIGO) guidelines (28), characterized by the presence of kidney structural or functional abnormalities for over 3 months and meeting at least one of the following criteria (28) (1): urinary albumin-to-creatinine ratio (UACR) ≥ 30 mg/g; (2) urinary sediment abnormalities; (3) histological or imaging evidence of structural kidney abnormalities; (4) history of kidney transplantation; or (5) eGFR < 60 mL/min/1.73 m². T2DM was defined by meeting any of the following criteria (29): (1) fasting blood glucose (FBG) ≥7.0 mmol/L; (2) 2-hour plasma glucose during an oral glucose tolerance test (OGTT) ≥ 11.1 mmol/L; (3) glycated hemoglobin (HbA1c) ≥ 6.5%; or (4) documented history of T2DM or current use of glucose-lowering medication. The exclusion criteria were as follows: (1) type 1 diabetes or other specific types of diabetes (e.g., steroid-induced diabetes); (2) primary glomerular diseases (e.g., IgA nephropathy) or other non-diabetes-related secondary glomerular diseases (e.g., lupus nephritis); (3) current renal replacement therapy (hemodialysis, peritoneal dialysis, or kidney transplantation); (4) comorbid severe cardiopulmonary dysfunction or history of malignancy; (5) presence of acute infection, pregnancy, or lactation; or (6) missing renal function data. A total of 994 patients were included in the final analysis. The study flowchart is presented in **Figure 1**.

### Data collection and definitions

Data were extracted from electronic medical records by uniformly trained researchers. Demographic and Clinical Characteristics: Age, sex, height, weight, blood pressure, duration of CKD, coronary heart disease (CHD), stroke, and hypertension (HBP). Laboratory Parameters: Fasting blood samples were collected for complete blood count, renal function, electrolytes, liver function, lipid profile, HbA1c, serum ferritin, etc. eGFR was calculated using the CKD-EPI 2021 equation (30). SII was calculated as platelet count × neutrophil count/lymphocyte count (12). Body Mass Index (BMI) was calculated as weight (kg)/height (m)². Hyperlipidemia was defined as total cholesterol ≥6.2 mmol/L, or LDL-C ≥4.1 mmol/L, or triglycerides ≥2.3 mmol/L (31). Anemia was defined as hemoglobin (HGB) <130 g/L for males and <120 g/L for females (32).

### Prolactin measurement

Serum PRL levels were measured as part of routine clinical laboratory testing in our hospital using an electrochemiluminescence immunoassay (ECLIA) on a Roche Cobas e 601 analyzer (Roche Diagnostics, Basel, Switzerland). Blood samples were collected under fasting conditions and analyzed within 24 hours of collection. PRL measurement was conducted in accordance with standardized operational procedures and internal quality control protocols. All reagent batches were validated for inter-batch consistency according to the manufacturer’s specifications and calibrated against the WHO International Standard 84/500. The inter-assay coefficient of variation (CV) remained <5% throughout the study period. The analytical measuring range was 1.00–10000 mIU/L. Reference ranges were 102–496 mIU/L for adult females and 66–324 mIU/L for adult males. Hyperprolactinemia was defined as PRL levels exceeding these upper limits. Throughout the study period, the same analytical platform and assay methodology were consistently used without changes in instrumentation or calibration standards.

### Statistical analysis

All analyses were performed using R software (version 4.3.2). Continuous variables are presented as mean ± standard deviation (SD) or median (interquartile range, IQR), and categorical variables as frequencies (percentages). Comparisons across PRL quartiles were conducted using one-way ANOVA or the Kruskal–Wallis test for continuous variables, and the chi-square test for categorical variables, as appropriate.

To evaluate the associations between serum PRL and clinical parameters, multivariable linear mixed-effects models were constructed and adjusted for age, sex, BMI, eGFR, HBP, CHD, and stroke. PRL was modeled as a continuous variable per 100 mIU/L increment. To investigate the relationship between PRL and the systemic SII, four models were constructed. The crude model included PRL only. Model 1 was adjusted for age, sex, eGFR, HBP, CHD, and stroke. Model 2 further included HbA1c and hyperlipidemia. Model 3 additionally adjusted for HGB and serum albumin. In these analyses, PRL was examined both as quartiles and as a continuous variable (per 100 mIU/L increment). All regression models were implemented as linear mixed-effects models with random intercepts for patient ID, thereby accounting for within-subject correlation arising from repeated measurements.

Restricted cubic spline (RCS) models were applied to explore potential nonlinear associations between serum PRL and the SII. PRL was modeled as a continuous variable using RCS with four knots placed at predefined percentiles. Models were adjusted for age, sex, eGFR, HBP, CHD, stroke, hyperlipidemia, HbA1c, HGB, and serum albumin. The predicted SII values displayed in the spline plots represent covariate-adjusted estimates derived from the fitted multivariable models. Nonlinearity was evaluated using likelihood ratio tests comparing models with and without spline terms. When a significant nonlinear association was identified, segmented regression analyses were performed to estimate slopes on either side of the inflection point.

Trend and nonlinearity tests were derived from regression models treating PRL as a continuous variable. Subgroup and sensitivity analyses were conducted to assess the robustness of the findings. Statistical significance was defined as a two-sided *p* < 0.05. Exact *p* values were reported wherever possible. Values less than 0.001 were presented in scientific notation. For results falling below the machine precision of double-precision floating-point arithmetic in R (.Machine$double.eps ≈ 2.22 × 10^-16^), *p* values were reported as *p* < 2.22 × 10^-16^.

## Results

### Baseline characteristics stratified by PRL quartiles

This cross-sectional analysis included 994 patients with T2DM-related DKD. As detailed in **Table 1**, the mean serum PRL concentration was 459.85 ± 692.70 mIU/L in the overall cohort, with a median concentration of 344.45 mIU/L (IQR: 258.80-463.15 mIU/L), suggesting a right-skewed distribution. When stratified by sex, the median PRL concentration was 363.00 mIU/L (IQR: 273.00-484.00) in females (n = 400) and 333.00 mIU/L (IQR: 249.00-454.00) in males (n = 594).

**Table 1:** Baseline characteristics of patients with diabetic kidney disease.

Variable	N	Overall (n = 994)	Quartile 1 (n = 249)	Quartile 2 (n = 249)	Quartile 3 (n = 248)	Quartile 4 (n = 248)	*p*-value
Prolactin (mIU/L)							
Mean (SD)	994	459.85 (692.70)	210.40 (37.50)	301.62 (24.08)	397.64 (34.63)	931.37 (1269.15)	< 2.22 × 10^-16^
Median (IQR)		344.45 (258.80-463.15)	217.70 (190.90-239.40)	302.70 (282.20-321.50)	393.50 (367.37-427.23)	598.10 (504.30-757.13)	
Demographics							
Age (years)	994	64.06 (10.72)	63.78 (9.57)	64.05 (9.64)	63.86 (10.99)	64.56 (12.46)	0.852
Sex n (%)	994						0.021
Female		400 (40.24%)	80 (32.13%)	102 (40.96%)	108 (43.55%)	110 (44.35%)	
Male		594 (59.76%)	169 (67.87%)	147 (59.04%)	140 (56.45%)	138 (55.65%)	
Body mass index (kg/m²)	945	25.72 (4.08)	25.87 (3.75)	26.15 (4.35)	25.59 (4.00)	25.24 (4.18)	0.099
Comorbidities							
Hypertension n (%)	994						0.008
YES		887 (89.24%)	214 (85.94%)	236 (94.78%)	221 (89.11%)	216 (87.10%)	
NO		107 (10.76%)	35 (14.06%)	13 (5.22%)	27 (10.89%)	32 (12.90%)	
Coronary heart disease n (%)	994						0.102
YES		236 (23.74%)	73 (29.32%)	58 (23.29%)	51 (20.56%)	54 (21.77%)	
NO		758 (76.26%)	176 (70.68%)	191 (76.71%)	197 (79.44%)	194 (78.23%)	
Stroke n (%)	990						0.519
YES		170 (17.17%)	49 (19.84%)	44 (17.81%)	38 (15.32%)	39 (15.73%)	
NO		820 (82.83%)	198 (80.16%)	203 (82.19%)	210 (84.68%)	209 (84.27%)	
Hyperlipidemia n (%)	994						0.370
YES		321 (32.29%)	85 (34.14%)	88 (35.34%)	77 (31.05%)	71 (28.63%)	
NO		673 (67.71%)	164 (65.86%)	161 (64.66%)	171 (68.95%)	177 (71.37%)	
Anemia n (%)	990						0.021
YES		596 (60.20%)	130 (52.63%)	151 (60.89%)	151 (61.13%)	164 (66.13%)	
NO		394 (39.80%)	117 (47.37%)	97 (39.11%)	96 (38.87%)	84 (33.87%)	
Renal Function & Proteinuria							
CKD stage n (%)	994						2.488 × 10^-6^
G1-G2		238 (23.94%)	67 (26.91%)	61 (24.50%)	60 (24.19%)	50 (20.16%)	
G3a-G3b		345 (34.71%)	111 (44.58%)	91 (36.55%)	79 (31.85%)	64 (25.81%)	
G4-G5		411 (41.35%)	71 (28.51%)	97 (38.96%)	109 (43.95%)	134 (54.03%)	
eGFR (mL/min/1.73 m²)	994	41.71 (28.85)	46.98 (25.99)	42.88 (26.98)	41.41 (29.53)	35.54 (31.59)	1.514 × 10^-4^
UACR (mg/g)	933	169.02 (1563.80)	92.83 (144.91)	112.19 (186.80)	155.96 (499.55)	326.22 (3161.89)	0.372
Urine Protein (g/24 h)	931	2347.83 (3573.71)	2041.59 (3132.01)	2244.30 (3512.94)	2561.13 (3901.97)	2557.14 (3706.84)	0.317
CKD Complication Parameters							
Hemoglobin (g/L)	990	117.06 (23.29)	122.80 (20.39)	118.30 (22.66)	116.66 (22.26)	110.51 (25.95)	7.795 × 10^-8^
Red blood cell (×10¹²/L)	991	3.97 (0.77)	4.16 (0.69)	4.01 (0.73)	3.95 (0.75)	3.77 (0.86)	4.688 × 10^-7^
Serum calcium (mmol/L)	981	2.22 (0.17)	2.24 (0.15)	2.23 (0.14)	2.22 (0.18)	2.19 (0.21)	0.025
Serum phosphorus (mmol/L)	981	1.30 (0.29)	1.22 (0.19)	1.27 (0.23)	1.30 (0.28)	1.39 (0.40)	1.434 × 10^-10^
iPTH (pg/mL)	516	83.28 (129.51)	54.61 (49.47)	63.53 (86.28)	72.37 (100.36)	141.56 (205.64)	4.265 × 10^-8^
Metabolic-related Parameters							
Glucose (mmol/L)	963	6.41 (2.42)	6.82 (2.52)	6.54 (2.60)	6.11 (2.20)	6.17 (2.30)	0.003
Hemoglobin A1c (%)	936	7.25 (1.47)	7.49 (1.51)	7.25 (1.36)	7.17 (1.56)	7.07 (1.43)	0.015
Alanine aminotransferase (U/L)	985	18.48 (16.56)	19.11 (13.51)	18.18 (12.93)	19.28 (22.51)	17.32 (15.48)	0.532
Immune-related Parameters							
White blood cell (×10^9^/L)	991	6.61 (2.00)	6.49 (1.93)	6.53 (1.69)	6.51 (2.06)	6.90 (2.25)	0.065
Neutrophil (×10^9^/L)	989	4.14 (1.68)	3.94 (1.53)	4.00 (1.24)	4.11 (1.80)	4.51 (2.00)	4.953 × 10^-4^
Lymphocyte (×10^9^/L)	991	1.79 (0.68)	1.88 (0.69)	1.85 (0.67)	1.73 (0.60)	1.71 (0.73)	0.010
Platelet (×10^9^/L)	991	213.73 (68.95)	210.26 (63.26)	211.54 (61.75)	212.36 (69.09)	220.76 (80.10)	0.314
Inflammation Parameters							
C-reactive protein (mg/L)	989	6.97 (16.46)	5.08 (8.91)	6.85 (19.40)	5.89 (9.92)	10.10 (22.79)	0.004
Serum ferritin (ng/mL)	558	273.52 (250.03)	290.19 (268.04)	284.65 (260.83)	239.59 (207.49)	283.99 (262.93)	0.281
Albumin (g/L)	986	38.21 (5.87)	38.73 (5.93)	38.60 (5.59)	38.23 (5.60)	37.27 (6.25)	0.025

Prolactin is presented both as mean (SD) and as median (IQR). All other continuous variables are presented as mean (SD); categorical variables as n (%). Overall *p*-values were calculated using one-way ANOVA for continuous variables and chi-square test (or Fisher’s exact test when appropriate). P-value reporting rules: Values less than 0.001 were presented in scientific notation. For results falling below the machine precision of double-precision floating-point arithmetic in R (.Machine$double.eps ≈ 2.22 × 10^-16^), *p* values were reported as *p* < 2.22 × 10^-16^. Sex-stratified prolactin levels: Female: Mean ± SD = 553.24 ± 1024.11; Median (IQR) = 362.80 (272.62-483.70); Male: Mean ± SD = 396.96 ± 296.58; Median (IQR) = 332.75 (248.80-454.42) Abbreviations: SD, standard deviation; IQR, interquartile range; CKD, chronic kidney disease; eGFR, estimated glomerular filtration rate; iPTH, intact parathyroid hormone; UACR, urinary albumin-to-creatinine ratio; IQR, interquartile range; SD, standard deviation.

Participants were stratified into quartiles (Quartile1-Quartile4) based on PRL levels. Significant differences in renal function were observed across quartiles, including a decline in eGFR (*p* = 1.5 × 10^-4^) and an increase in the proportion of advanced CKD (G4-G5), rising from 29% in Quartile1 to 54% in Quartile4 (*p* = 2.5 × 10^-6^). Markers related to CKD complications differed significantly across PRL quartiles. HGB and red blood cell (RBC) decreased across higher PRL quartiles (*p* = 7.8 × 10–^8^ and *p* = 4.7 × 10^-7^, respectively), while serum phosphorus and intact parathyroid hormone (iPTH) increased (*p* = 1.4 × 10–^10^ and *p* = 4.3×10^-8^, respectively). Serum albumin was lower in the highest PRL quartile (*p* = 0.025). Glucose and HbA1c levels were lower across increasing PRL quartiles (*p* = 0.003 and *p* = 0.015, respectively). Among inflammation markers, neutrophil count increased and lymphocyte count decreased across PRL quartiles (*p* = 5 × 10–^4^ and *p*= 0.01, respectively), whereas no significant differences were found in CRP or platelet levels. PRL levels were associated with sex distribution (*p* = 0.021) but not with age, BMI, or history of stroke or hyperlipidemia.

### Associations between serum PRL and clinical parameters

Of the 994 observations, 684 individuals contributed repeated measurements (mean 1.43 visits per subject; 137 with ≥2 visits). To account for within-subject clustering, associations between serum PRL and clinical parameters were evaluated using linear mixed-effects models with random intercepts for patient ID. PRL was modeled as a continuous variable per 100 mIU/L increment.

As shown in **Table 2**, in the multivariable mixed-effects model adjusted for sex, age, BMI, eGFR, hypertension, stroke, and CHD, higher PRL levels were associated with several inflammation-related markers of inflammation and immune activation, including increased white blood cell count (β = 0.035, 95% CI: 0.019 to 0.050; *p* = 1.29 × 10^-5^), neutrophil count (β = 0.033, 95% CI: 0.019 to 0.047; *p* = 5.18 × 10^-6^), serum ferritin (β = 3.728, 95% CI: 2.011 to 5.444; *p* = 3.32 × 10^-5^), as well as decreased serum albumin levels (β = -0.043, 95% CI: -0.083 to -0.004; *p* = 0.032). Positive associations were also observed for CKD-mineral and bone disorder markers, including serum phosphorus (β = 0.003, 95% CI: 0.001 to 0.006; *p* = 0.015) and iPTH (β = 2.295, 95% CI: 0.586 to 4.003; *p* = 0.009). In contrast, the previously observed univariate associations with HGB and red blood cell count were no longer statistically significant after multivariable adjustment.

**Table 2:** Univariate and multivariate mixed model analysis of association between serum PRL (per 100 mIU/L Increase) and clinical parameters.

	Univariate mixed model		Multivariate mixed model	
Outcome variable	β (95% CI)	*p*-value	β (95% CI)	*p*-value
Hemoglobin (g/L)	-0.162 (-0.298, -0.026)	0.020	-0.052 (-0.179, 0.074)	0.416
Red blood cell (×10¹²/L)	-0.007 (-0.011, -0.002)	0.005	-0.002 (-0.007, 0.002)	0.265
White blood cell (×10^9^/L)	0.035 (0.020, 0.050)	4.25 × 10^-6^	0.035 (0.019, 0.050)	1.29 × 10^-5^
Platelet (×10^9^/L)	0.255 (-0.138, 0.647)	0.203	0.367 (-0.044, 0.777)	0.080
Neutrophil (×10^9^/L)	0.036 (0.023, 0.050)	1.98 × 10^-7^	0.033 (0.019, 0.047)	5.18 × 10^-6^
Lymphocyte (×10^9^/L)	-0.003 (-0.008, 0.001)	0.126	-0.002 (-0.006, 0.003)	0.496
C-reactive protein (mg/L)	0.060 (-0.035, 0.156)	0.214	0.043 (-0.057, 0.143)	0.395
Serum ferritin (ng/mL)	3.465 (1.791, 5.139)	7.14 × 10^-5^	3.728 (2.011, 5.444)	3.32 × 10^-5^
Albumin (g/L)	-0.063 (-0.101, -0.025)	0.001	-0.043 (-0.083, -0.004)	0.032
Alanine aminotransferase (U/L)	-0.151 (-0.305, 0.003)	0.054	-0.103 (-0.259, 0.053)	0.194
Glucose (mmol/L)	0.020 (-0.003, 0.043)	0.089	0.022 (-0.001, 0.045)	0.062
Hemoglobin A1c (%)	0.007 (-0.004, 0.017)	0.215	0.009 (-0.002, 0.020)	0.091
Serum calcium (mmol/L)	-0.001 (-0.002, 0.000)	0.184	-0.001 (-0.002, 0.001)	0.307
Serum phosphorus (mmol/L)	0.005 (0.003, 0.008)	1.28 × 10^-4^	0.003 (0.001, 0.006)	0.015
iPTH (pg/mL)	4.304 (2.501, 6.106)	3.72 × 10^-6^	2.295 (0.586, 4.003)	0.009
UACR (mg/g)	-0.036 (-1.498, 1.426)	0.962	-0.548 (-2.164, 1.067)	0.504
Urine Protein (g/24h)	-5.151 (-30.269, 19.968)	0.687	-12.966 (-34.908, 8.975)	0.246

Univariate mixed model: linear mixed-effects model with PRL modeled per 100 mIU/L increment and random intercept for each patient. Multivariate mixed model: multivariable linear mixed-effects model adjusted for sex, age (years), BMI (kg/m²), eGFR (mL/min/1.73 m²), hypertension, stroke, and coronary heart disease, with random intercept for each patient. All models account for repeated measurements within patients using random intercepts. P values correspond to two-sided t-tests for the PRL term in each model (Satterthwaite approximation) and were reported in scientific notation when < 0.001. Data are presented as β coefficients with 95% confidence intervals (CI). PRL, prolactin; CI, confidence interval; iPTH, intact parathyroid hormone; UACR, urinary albumin-to-creatinine ratio.

### Association between PRL levels and the SII

As shown in **Table 3**, both quartile-based and continuous analyses using mixed-effects models with random intercepts for patient ID revealed a significant and robust positive association between PRL and SII. In the fully adjusted model (Model 3), compared with the lowest Quartile 1, the β coefficients (95% CI) for SII were 87.00 (2.84 to 171.16) in Quartile 3 and 197.85 (105.52 to 290.18) in Quartile 4, with a significant trend across ascending PRL quartiles (*p* for trend = 1.55 × 10^-5^). Consistent results were observed in the continuous analysis. In Model 3, each 100 mIU/L increase in PRL was associated with a 7.10-unit increase in SII (95% CI: 3.13 to 11.07; *p* = 5.12 × 10^-4^).

**Table 3:** Association between prolactin and SII.

	PRL quartiles analysis					PRL continuous analysis	
	Quartile 1 (n=249)	Quartile 2 (n=249)	Quartile 3 (n=248)	Quartile 4 (n=248)	*p* for trend	Overall (per 100 mIU/L) (N = 994)	*p*-value
Prolactin (mIU/L)	217.70 (190.90, 239.40)	302.70 (282.20, 321.50)	393.50 (367.37, 427.23)	598.10 (504.30, 757.13)		344.45 (258.80, 463.15)	
Crude model	1 (Ref)	39.34 (-37.89, 116.56)	116.00 (35.40, 196.60)	254.15 (167.67, 340.64)	4.41 × 10^-9^	8.20 (4.50, 11.90)	1.69 × 10^-5^
Model 1	1 (Ref)	25.88 (-51.43, 103.19)	92.67 (12.24, 173.10)	207.65 (120.53, 294.78)	1.50 × 10^-6^	7.13 (3.45, 10.80)	1.63 × 10^-4^
Model 2	1 (Ref)	23.62 (-58.07, 105.31)	89.11 (4.50, 173.71)	215.04 (122.53, 307.55)	3.04 × 10^-6^	7.71 (3.74, 11.68)	1.64 × 10^-4^
Model 3	1 (Ref)	23.87 (-57.59, 105.33)	87.00 (2.84, 171.16)	197.85 (105.52, 290.18)	1.55 × 10^-5^	7.10 (3.13, 11.07)	5.12 × 10^-4^

Main analysis: Association between prolactin and SII in the complete study population. A total of 994 measurements from 684 individuals were included. Data are presented as β coefficients (95% confidence interval) for SII. PRL quartiles analysis: Values are median (interquartile range) for prolactin levels in mIU/L for each quartile (Quartile 1–4). The overall median (IQR) for prolactin levels is 344.45 (258.80, 463.15) mIU/L. SII: median (IQR) = 455.51 (325.76–671.19); mean ± SD = 575.41 ± 502.04. PRL continuous analysis: β coefficients represent the change in SII per 100 mIU/L increase in prolactin. Crude model: unadjusted linear mixed-effects model with random intercept for individual ID. Model 1: adjusted for age, sex, eGFR, and history of hypertension, coronary heart disease, and stroke, with random intercept for individual ID. Model 2: Model 1 + hyperlipidemia and HbA1c. Model 3: Model 2 + albumin and hemoglobin. All models are linear mixed-effects models with random intercepts for individual ID to account for repeated measures. P values correspond to two-sided tests for the PRL term in each model and were reported in scientific notation when < 0.001. Abbreviations: ref, reference; SII, systemic immune-inflammation index; PRL, prolactin; ID, patient identification.

### Nonlinear association and threshold effect between PRL and SII

**Figure 2:**
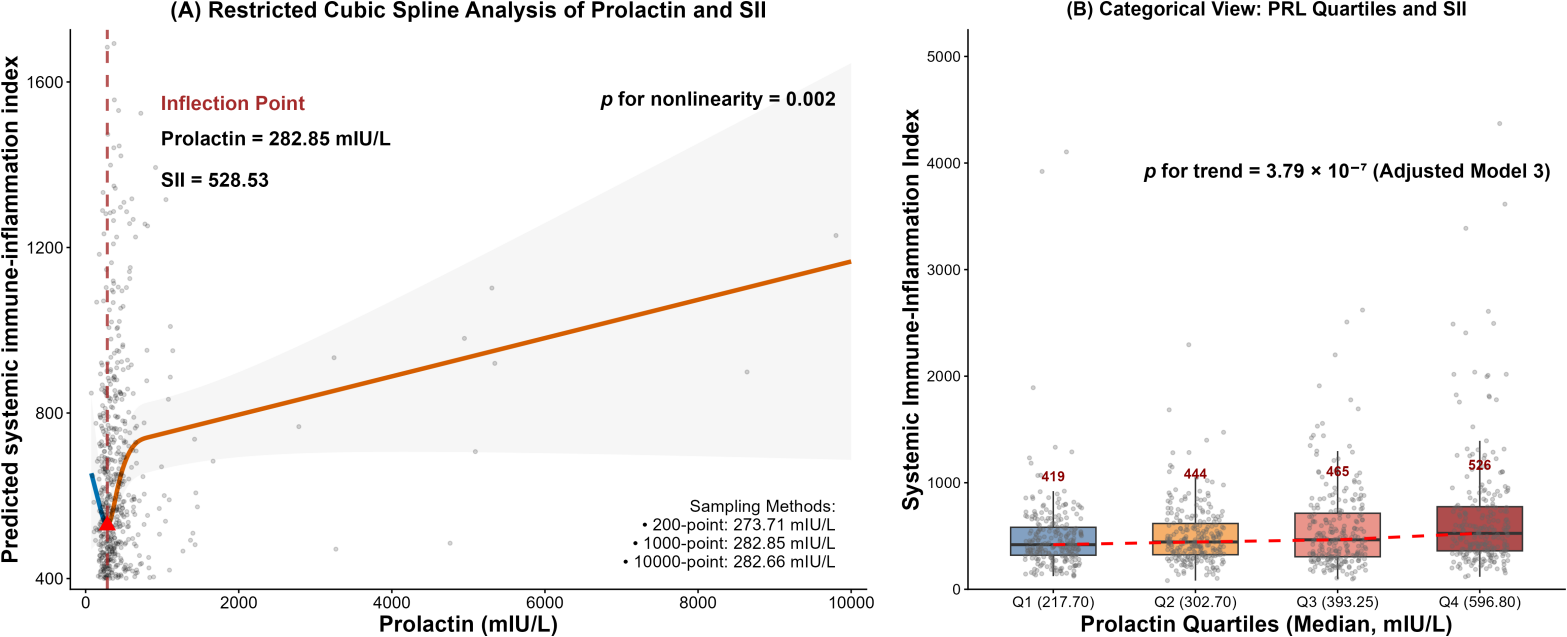
Association between serum PRL and the SII. **(A)** Restricted cubic spline (RCS) analysis showing the adjusted association between serum PRL and SII in the overall cohort. Shaded areas indicate 95% confidence intervals. The vertical dashed line marks the estimated inflection point, and the red triangle indicates the predicted minimum SII. **(B)** Distribution of SII across PRL quartiles. Boxes represent interquartile ranges, horizontal lines indicate medians, dashed lines connect median values across quartiles, and red numbers denote median SII values. Abbreviations: SII, systemic immune-inflammation index; PRL, prolactin; RCS, restricted cubic spline; eGFR, estimated glomerular filtration rate; HbA1c, hemoglobin A1c.

To further explore the nonlinear relationship between serum PRL and the SII, we performed RCS analyses. The spline model revealed a statistically significant nonlinear association (*p* for nonlinearity = 0.002), with an inflection point identified at 282.85 mIU/L (**Figure 2A**). Consistent with the continuous spline analysis, categorical analyses based on PRL quartiles showed a progressive increase in SII across quartiles (**Figure 2B**). Median SII values increased stepwise from Quartile 1 to Quartile 4, and the overall trend across quartiles was statistically significant (*p* for trend = 3.79 × 10^-7^). Segmented regression analysis based on the spline curve indicated that the association between PRL and SII differed markedly across PRL concentration ranges (**Supplementary Table S1**). Specifically, in the segments below the inflection point, the slopes were negative (β = -0.65, *p <* 2.22 × 10^-16^). However, when PRL concentrations exceeded 282.85 mIU/L, the association reversed to a positive direction (β = 0.48, *p <* 2.22 × 10^-16^) and remained positive in the highest concentration segment (≥ 769.70 mIU/L, β = 0.05, *p <* 2.22 × 10^-16^).

### Sensitivity and subgroup analyses

We performed systematic subgroup and sensitivity analyses to validate the robustness of our findings. Stratified analysis (**Supplementary Table S2**) showed that the positive association between PRL and SII was significantly stronger in subgroups of patients aged ≥ 65 years (*p* for interaction = 7.02 × 10^-4^), those with advanced CKD (G4–G5; *p* for interaction = 0.003), and those with HBP (*p* for interaction = 0.007). In contrast, no significant effect modification was observed in subgroups stratified by sex, CHD, Stroke, BMI, hyperlipidemia, or anemia status (all *p* for interaction > 0.05).

To further assess the robustness of the primary findings, a series of sensitivity analyses were conducted. After excluding patients with conditions directly affecting PRL (pituitary adenoma or dopaminergic drug use; n = 965) or those with potential acute inflammation (CRP > 50 mg/L; n = 970), the positive association remained significant, with effect estimates highly consistent with the main analysis (β = 6.930 and β = 6.855, respectively; **Supplementary Tables S3**, **S4**). Notably, the magnitude of the continuous association increased markedly after excluding patients with extreme PRL levels (4240 mIU/L) and without confirmatory MRI (β = 27.230, 95% CI: 14.394 to 40.066; *p* = 3.44 × 10^-5^; **Supplementary Table S5**).

## Discussion

To our knowledge, this study provided the first evidence that serum PRL was independently and dose-dependently associated with SII in type 2 DKD. After adjusting for multiple confounders, every 100 mIU/L increment in PRL corresponded to a 7.10-unit rise in SII (95% CI 3.1 to 11.1). RCS analysis disclosed a significant nonlinear association, with an inflection point at 282.85 mIU/L. Below this concentration, PRL was inversely related to SII, whereas above it the relationship switched to a steep positive dose-response (*p* for nonlinearity = 0.002). The overall association remained consistent across sex, CKD stage, and medication subgroups and survived a battery of sensitivity analyses that sequentially excluded individuals with pituitary adenomas, dopamine-agonist or oestrogen use, extreme outlying values, or evidence of acute inflammation, underscoring the robustness of the observation.

The SII integrates three key cellular arms of innate immunity—neutrophils, platelets, and lymphocytes—into a single quantitative metric, providing a measure that more faithfully reflects the net balance between inflammatory activity and immune competence than any individual blood cell count alone (11, 12). Its prognostic value has been validated across a spectrum of pathologies, including solid tumors, autoimmune encephalitis, systemic lupus erythematosus, adult-onset Still’s disease, acute pancreatitis, ulcerative colitis, chronic obstructive pulmonary disease, and CHD (13–17, 33–35). It is also used to assess the incidence and severity of CHD (18, 36, 37). Recent cohort studies have extended this repertoire to DKD, demonstrating that higher SII portends faster eGFR decline and greater cardiovascular risk (19–21). In the present cohort, neutrophil counts rose monotonically across PRL quartiles, whereas lymphocyte counts exhibited the opposite trend (**Table 1**). Multivariable-adjusted regression revealed that each 100 mIU/L increment in serum PRL was accompanied by a measurable increase in both neutrophil and white blood cell counts (**Table 2**). These data corroborate the existence of a sub-clinical, immune-mediated inflammatory milieu in DKD and underscore the utility of SII as an integrative marker of systemic inflammation in this population.

Renal clearance normally accounts for ≥80% of PRL elimination (38). Consequently, hyperprolactinemia is usually interpreted as an epiphenomenon of declining glomerular filtration rather than a pathogenic mediator in CKD (39, 40). However, high PRL levels can directly activate immune cells via the PRL receptor, triggering the JAK/STAT and MAPK signaling pathways, promoting macrophage activation, and enhancing the synthesis and secretion of pro-inflammatory cytokines, thereby exacerbating microvascular endothelial dysfunction (41, 42). In this context, our cross-sectional findings suggest that elevated PRL may not merely reflect impaired renal clearance but may also be linked to the accompanying immune-inflammatory milieu in DKD. Nevertheless, given the observational design of the study, the directionality and potential causal role of PRL require confirmation in longitudinal and mechanistic investigations.

As a pleiotropic hormone, PRL exerts potent immunomodulatory functions through its receptors widely expressed on immune cells (22, 23). This biology predicts a biphasic dose-response curve: nanomolar concentrations promote thymocyte survival, immunoglobulin class-switching, and regulatory T-cell expansion, whereas supra-physiological concentrations activate the JAK2/STAT5 and MAPK axes, generate reactive oxygen species, and release neutrophil extracellular traps (22, 23). Our observed nonlinear pattern is compatible with prior experimental evidence suggesting a biphasic immunomodulatory profile of PRL. RCS analysis identified an inflection point at 282.85 mIU/L. Below this threshold, PRL correlated inversely with the SII (**Table S1**), consistent with a homeostatic immunomodulatory role. Above the threshold, however, the association shifted to a significant positive correlation, a transition accompanied by progressive increases in neutrophil and white blood cell counts that remained independent of glycemic control, blood pressure, and renal function. Notably, the cohort mean (459.85 mIU/L, **Table 1**) lay well above the inflection point, indicating that the majority of participants were already in the higher PRL range where a positive PRL–SII association was observed. The identified inflection point at 282.85 mIU/L suggests a potential shift in the PRL-SII relationship. While this exploratory finding could be interpreted as a tentative quantitative boundary distinguishing between different immunological phases of PRL activity, it cannot be considered a definitive clinical threshold without external validation.

Like CRP, PRL rises early in acute insults such as viral infections (e.g., COVID-19), sepsis, or systemic inflammatory response syndrome (43). In the present cohort, however, the higher PRL quartiles were characterized by lower, not higher, fasting glucose and HbA1c, and multivariable modelling stripped away any residual glycemic signal. Instead, PRL was positively associated with white-cell count, neutrophils, ferritin, and albumin─cellular and iron-inflammatory indices─while remaining independent of CRP (**Tables 1**, **2**). The PRL–SII association was most pronounced in patients ≥ 65 years or those with HBP or CHD, conditions themselves defined by chronic low-grade inflammation (44, 45). These data indicate that DKD patients exhibit a chronic low-grade inflammatory state distinct from acute infection.

To ensure the reliability of our conclusions, we conducted a series of subgroup and sensitivity analyses. The independent association between PRL and SII was consistently observed across various subgroups and remained unchanged after excluding potential biases. Furthermore, the persistence of this dose-response relationship in patients with advanced CKD (stage G4-G5) supports the notion that the link between PRL and inflammation may remain relevant even in the late stages of DKD. Strengths of our study include its single-center design, which ensured standardized measurements, and the use of robust statistical methodologies, including linear mixed-effects models and RCS analysis. However, our study has limitations. First, its cross-sectional design precludes the inference of causality. Second, we measured total PRL without distinguishing between different bioactive isoforms, such as macroprolactin, which could affect the precision of our results. Third, the lack of kidney biopsy data prevents a direct investigation of the relationship between PRL levels and specific types of renal tissue injury.

## Conclusion

In conclusion, this cross-sectional study demonstrates an independent, non-linear association between serum PRL levels and the SII in patients with T2DM-related DKD. An inflection point was identified at 282.85 mIU/L, Below this threshold, PRL was inversely associated with SII, whereas above it the association became positive, suggesting a shift in the PRL–SII relationship across different concentration ranges. This exploratory finding suggests a potential link between PRL and dysregulation of the immune–inflammatory axis in DKD, which warrants further mechanistic and longitudinal investigation.

## Data Availability

The original contributions presented in the study are included in the article/**Supplementary Material**. Further inquiries can be directed to the corresponding authors.
