# Supplementary material for: Correlation between serum prolactin and the systemic immune-inflammation index in diabetic kidney disease: a cross-sectional study
Source: Front Endocrinol (Lausanne). 2026 Mar 2;17:1772810. doi: 10.3389/fendo.2026.1772810 (PMC12989830; doi:10.3389/fendo.2026.1772810)
Supplement: Supplementary file 1 [file Table1.docx]

**Supplementary Tables**

**TABLE S1:** Segmented Linear Mixed-Effects Model Analysis of PRL and SII Association

**TABLE S2:** Stratified Analysis: Association between Prolactin Quartiles and SII - Mixed Model 3

**TABLE S3:** Sensitivity Analysis 1: Association between Prolactin and SII

**TABLE S4**: Sensitivity Analysis 2: Association between Prolactin and SII

**TABLE S5:** Sensitivity Analysis 3: Association between Prolactin and SII

**TABLE S1 Segmented Linear Mixed-Effects Model Analysis of PRL and SII Association***^1^*

| Segment | Phase | PRL Range  (mIU/L) | Slope  (per mIU/L PRL) | Slope  (per 100 mIU/L PRL) | *p*-value | Clinical  Interpretation |
| --- | --- | --- | --- | --- | --- | --- |
| **1** | Before inflection point | 74.20-282.85 | -0.65 | -65.00 | < 2.22 × 10⁻¹⁶ | Negative |
| **2** | After inflection point, Stage 1 | 282.85-759.77 | 0.48 | 48.10 | < 2.22 × 10⁻¹⁶ | Positive |
| **3** | After inflection point, Stage 2 | 769.70-10000.00 | 0.05 | 4.60 | < 2.22 × 10⁻¹⁶ | Positive |
| *^1^*Segmented linear mixed-effects model analysis of the association between prolactin (PRL) and Systemic Immune-Inflammation Index (SII). Three distinct segments were identified based on piecewise linear regression: 1) Before inflection point (74.20-282.85 mIU/L): PRL shows a negative association with SII. 2) After inflection point, Stage 1 (282.85-759.77 mIU/L): PRL shows a positive association with SII. 3) After inflection point, Stage 2 (769.70-10000.00 mIU/L): PRL shows a weak positive association with SII. Slope represents the change in SII per unit change in PRL. Slope per 100 represents the change in SII per 100 mIU/L change in PRL. All models are linear mixed-effects models with random intercepts for individual ID to account for repeated measures. The inflection point was determined using piecewise linear regression with maximum likelihood estimation. P-value reporting rules:values between 1×10⁻¹⁶ and 0.001 are reported in scientific notation; Values <1×10⁻¹⁶ are reported as <1×10⁻¹⁶. Abbreviations: SII, Systemic Immune-Inflammation Index; PRL, Prolactin; ID, Identification | | | | | | |

**TABLE S2 Stratified Analysis: Association between Prolactin Quartiles and SII - Mixed Model 3***^1^*

|  | **Prolactin Quartiles** | | | |  |  |
| --- | --- | --- | --- | --- | --- | --- |
|  | Quartile 1 | Quartile 2 | Quartile 3 | Quartile 4 | *p* for trend | *p* for interaction |
| *Prolactin (mIU/L)* | 217.7 (190.9, 239.4) | 302.7 (282.2, 321.5) | 393.5 (367.4, 427.2) | 598.1 (504.3, 757.1) |  |  |
| Sex |  |  |  |  |  | 0.986 |
| Female | 1 (Ref) | 32.74 (-69.59, 135.08) | 95.23 (-14.54, 205.01) | 244.03 (120.71, 367.35) | 1.40 × 10⁻⁴ |  |
| Male | 1 (Ref) | 13.04 (-99.65, 125.74) | 63.06 (-53.74, 179.86) | 177.23 (53.43, 301.02) | 0.005 |  |
| Age |  |  |  |  |  | 7.02 × 10⁻⁴ |
| <65 | 1 (Ref) | -12.97 (-132.10, 106.16) | 74.88 (-49.25, 199.01) | 41.73 (-94.42, 177.87) | 0.318 |  |
| ≥65 | 1 (Ref) | 37.62 (-68.03, 143.28) | 94.08 (-19.76, 207.93) | 336.14 (211.99, 460.30) | 5.13 × 10⁻⁷ |  |
| HBP |  |  |  |  |  | 0.007 |
| NO | 1 (Ref) | 50.05 (-147.13, 247.23) | 278.66 (-9.84, 567.16) | -9.07 (-295.77, 277.64) | 0.404 |  |
| YES | 1 (Ref) | 20.85 (-63.38, 105.08) | 53.57 (-32.70, 139.83) | 238.72 (144.20, 333.23) | 2.52 × 10⁻⁶ |  |
| CHD |  |  |  |  |  | 0.683 |
| NO | 1 (Ref) | -11.62 (-111.68, 88.45) | 67.87 (-36.39, 172.13) | 161.37 (47.20, 275.55) | 0.003 |  |
| YES | 1 (Ref) | 62.87 (-60.64, 186.37) | 106.07 (-16.81, 228.95) | 273.95 (127.84, 420.06) | 3.29 × 10⁻⁴ |  |
| Stroke |  |  |  |  |  | 0.330 |
| NO | 1 (Ref) | 28.79 (-60.19, 117.76) | 72.53 (-18.77, 163.83) | 217.91 (118.24, 317.59) | 2.60 × 10⁻⁵ |  |
| YES | 1 (Ref) | -26.93 (-228.54, 174.68) | 121.00 (-90.71, 332.71) | 128.73 (-127.67, 385.14) | 0.179 |  |
| CKD Stage |  |  |  |  |  | 0.003 |
| G1-G2 | 1 (Ref) | 4.06 (-85.71, 93.83) | 30.15 (-57.55, 117.86) | 7.37 (-84.09, 98.82) | 0.744 |  |
| G3a-G3b | 1 (Ref) | -21.54 (-123.74, 80.66) | -7.96 (-110.10, 94.17) | 77.99 (-31.52, 187.49) | 0.197 |  |
| G4-G5 | 1 (Ref) | 52.33 (-112.37, 217.03) | 213.26 (32.95, 393.57) | 336.76 (128.94, 544.58) | 7.04 × 10⁻⁴ |  |
| BMI |  |  |  |  |  | 0.518 |
| <25 | 1 (Ref) | 34.39 (-87.94, 156.72) | 122.71 (-2.15, 247.58) | 229.11 (83.87, 374.35) | 0.001 |  |
| ≥25 | 1 (Ref) | -32.06 (-128.53, 64.42) | -4.46 (-102.57, 93.65) | 113.45 (11.51, 215.39) | 0.029 |  |
| HLP |  |  |  |  |  | 0.540 |
| NO | 1 (Ref) | 28.04 (-74.25, 130.33) | 144.48 (36.88, 252.09) | 238.08 (119.49, 356.68) | 1.97 × 10⁻⁵ |  |
| YES | 1 (Ref) | -10.85 (-138.58, 116.87) | -15.59 (-142.58, 111.41) | 99.13 (-34.05, 232.30) | 0.195 |  |
| Anemia |  |  |  |  |  | 0.693 |
| NO | 1 (Ref) | 51.77 (-41.83, 145.37) | 90.11 (-7.81, 188.04) | 163.27 (53.98, 272.56) | 0.003 |  |
| YES | 1 (Ref) | 23.17 (-93.74, 140.08) | 128.06 (3.14, 252.98) | 228.94 (94.15, 363.72) | 3.68 × 10⁻⁴ |  |
| *^1^*Data are presented as β coefficients (95% confidence interval) for SII. Values are median (interquartile range) for prolactin (mIU/L). Mixed Model 3: adjusted for Age (years), Sex (male/female), HLP (yes/no), HbA1c (%), eGFR (ml/min/1.73 m²), HBP (yes/no), CHD (yes/no), Stroke (yes/no), ALB (g/L), HGB (g/L) with random intercept for ID. All models are linear mixed-effects models with random intercepts for individual ID to account for repeated measures. P values correspond to two-sided tests and were reported in scientific notation when < 0.001. Abbreviations: ref, reference; SII, Systemic Immune-Inflammation Index; PRL, prolactin; HBP, hypertension; HLP, hyperlipidemia; CHD, coronary heart disease; HbA1c, hemoglobin A1c; eGFR, estimated glomerular filtration rate; ALB, albumin; HGB, hemoglobin; CKD: Chronic kidney disease; ID, patient identification. | | | | | | |

**TABLE S3 Sensitivity Analysis 1: Association between Prolactin and SII*^1^***

|  | **PRL Quartiles Analysis** | | | | | **PRL Continuous Analysis** | |
| --- | --- | --- | --- | --- | --- | --- | --- |
|  | Quartile 1  (n=242) | Quartile 2  (n=241) | Quartile 3  (n=241) | Quartile 4  (n=241) | *p* for trend | Overall (per 100 mIU/L)  (N=965) | *p*-value |
| **Prolactin (mIU/L)** | 217.50 (188.93, 239.10) | 299.50 (279.30, 319.20) | 387.90 (364.30, 421.80) | 576.20 (497.20, 724.40) |  | 340.10 (258.20, 458.60) |  |
| **Crude model** | 1 (Ref) | 35.82 (-42.68, 114.32) | 105.19 (23.17, 187.22) | 263.06 (174.64, 351.47) | 6.31 × 10⁻⁹ | 8.11 (4.34, 11.88) | 3.06 × 10⁻⁵ |
| **Model 1** | 1 (Ref) | 27.49 (-50.96, 105.93) | 83.76 (1.91, 165.61) | 218.40 (129.32, 307.48) | 1.70 × 10⁻⁶ | 7.03 (3.28, 10.77) | 2.66 × 10⁻⁴ |
| **Model 2** | 1 (Ref) | 25.20 (-57.67, 108.07) | 80.37 (-5.81, 166.55) | 227.33 (132.66, 322.00) | 3.20 × 10⁻⁶ | 7.56 (3.51, 11.61) | 2.85 × 10⁻⁴ |
| **Model 3** | 1 (Ref) | 25.04 (-57.57, 107.66) | 77.71 (-7.93, 163.35) | 212.80 (118.55, 307.06) | 1.16 × 10⁻⁵ | 6.93 (2.88, 10.98) | 8.81 × 10⁻⁴ |
| *^1^*Sensitivity Analysis 1: Excluding patients with pituitary adenoma or using dopaminergic drugs. A total of 965 measurements from 667 individuals were included. 29 measurements were excluded from the original dataset. Data are presented as β coefficients (95% confidence interval) for SII. PRL quartiles analysis: Values are median (interquartile range) for prolactin levels in mIU/L for each quartile (Quartile 1–4). The overall median (IQR) for prolactin levels is 340.10 (258.20, 458.60) mIU/L. SII: median (IQR) = 457.58 (325.59–679.16); mean ± SD = 577.59 ± 505.80. PRL continuous analysis: β coefficients represent the change in SII per 100 mIU/L increase in prolactin. Crude model: unadjusted linear mixed-effects model with random intercept for individual ID. Model 1: adjusted for age, sex, eGFR, and history of hypertension, coronary heart disease, and stroke, with random intercept for individual ID. Model 2: Model 1 + hyperlipidemia and HbA1c. Model 3: Model 2 + albumin and hemoglobin. All models are linear mixed-effects models with random intercepts for individual ID to account for repeated measures. P values correspond to two-sided tests for the PRL term in each model and were reported in scientific notation when < 0.001. Abbreviations: ref, reference; SII, systemic immune-inflammation index; PRL, prolactin; ID, patient identification. | | | | | | | |

**TABLE S4 Sensitivity Analysis 2: Association between Prolactin and SII*^1^***

|  | **PRL Quartiles Analysis** | | | | | **PRL Continuous Analysis** | |
| --- | --- | --- | --- | --- | --- | --- | --- |
|  | Quartile 1  (n=243) | Quartile 2  (n=243) | Quartile 3  (n=242) | Quartile 4  (n=242) | *p* for trend | Overall (per 100 mIU/L)  (N=970) | *p*-value |
| **Prolactin (mIU/L)** | 216.70 (189.35, 239.10) | 300.10 (280.70, 320.05) | 392.30 (366.17, 423.38) | 584.35 (500.18, 742.67) |  | 343.10 (258.45, 459.40) |  |
| **Crude model** | 1 (Ref) | 26.44 (-37.67, 90.55) | 86.91 (21.02, 152.80) | 189.13 (119.92, 258.34) | 3.59 × 10⁻⁸ | 7.88 (4.62, 11.13) | 2.55 × 10⁻⁶ |
| **Model 1** | 1 (Ref) | 16.23 (-47.39, 79.85) | 71.58 (6.30, 136.87) | 161.64 (92.86, 230.43) | 1.71 × 10⁻⁶ | 7.15 (3.94, 10.37) | 1.51 × 10⁻⁵ |
| **Model 2** | 1 (Ref) | 14.86 (-52.18, 81.90) | 67.74 (-0.91, 136.38) | 161.61 (88.63, 234.59) | 6.99 × 10⁻⁶ | 7.45 (3.96, 10.94) | 3.32 × 10⁻⁵ |
| **Model 3** | 1 (Ref) | 12.16 (-54.37, 78.70) | 63.53 (-4.31, 131.38) | 146.20 (73.88, 218.52) | 3.61 × 10⁻⁵ | 6.85 (3.37, 10.34) | 1.27 × 10⁻⁴ |
| *^1^*Sensitivity Analysis 2: Excluding patients with C-reactive protein (CRP) > 50 mg/L to reduce the influence of acute inflammation. A total of 970 measurements from 663 individuals were included. 24 measurements were excluded from the original dataset (CRP > 50 mg/L). In the included sample, the median (IQR) CRP level was 3.23 (3.02, 3.40) mg/L (non-missing n=965). Data are presented as β coefficients (95% confidence interval) for SII. PRL quartiles analysis: Values are median (interquartile range) for prolactin levels in mIU/L for each quartile (Quartile 1–4). The overall median (IQR) for prolactin levels is 343.10 (258.45, 459.40) mIU/L. SII: median (IQR) = 446.65 (323.39–655.85); mean ± SD = 544.99 ± 389.52. PRL continuous analysis: β coefficients represent the change in SII per 100 mIU/L increase in prolactin. Crude model: unadjusted linear mixed-effects model with random intercept for individual ID. Model 1: adjusted for age, sex, eGFR, and history of hypertension, coronary heart disease, and stroke, with random intercept for individual ID. Model 2: Model 1 + hyperlipidemia and HbA1c. Model 3: Model 2 + albumin and hemoglobin. All models are linear mixed-effects models with random intercepts for individual ID to account for repeated measures. P values correspond to two-sided tests for the PRL term in each model and were reported in scientific notation when < 0.001. Abbreviations: ref, reference; SII, systemic immune-inflammation index; PRL, prolactin; CRP, C-reactive protein; ID, patient identification. | | | | | | | |

**TABLE S5 Sensitivity Analysis 3: Association between Prolactin and SII*^1^***

|  | **PRL Quartiles Analysis** | | | | | **PRL Continuous Analysis** | |
| --- | --- | --- | --- | --- | --- | --- | --- |
|  | Quartile 1  (n=247) | Quartile 2  (n=246) | Quartile 3  (n=246) | Quartile 4  (n=246) | *p* for trend | Overall (per 100 mIU/L)  (N=985) | *p*-value |
| **Prolactin (mIU/L)** | 217.30 (190.55, 239.15) | 301.00 (280.65, 320.32) | 390.40 (365.28, 422.88) | 575.45 (497.52, 709.30) |  | 341.30 (258.60, 459.10) |  |
| **Crude model** | 1 (Ref) | 42.68 (-33.44, 118.79) | 107.59 (27.46, 187.71) | 243.98 (156.00, 331.96) | 5.58 × 10⁻⁸ | 33.68 (22.23, 45.14) | 1.10 × 10⁻⁸ |
| **Model 1** | 1 (Ref) | 28.64 (-47.63, 104.90) | 83.26 (3.24, 163.27) | 199.54 (111.14, 287.94) | 8.82 × 10⁻⁶ | 26.33 (14.84, 37.82) | 7.91 × 10⁻⁶ |
| **Model 2** | 1 (Ref) | 25.63 (-54.89, 106.14) | 78.12 (-6.05, 162.30) | 204.38 (110.83, 297.92) | 1.83 × 10⁻⁵ | 29.37 (16.74, 42.01) | 5.93 × 10⁻⁶ |
| **Model 3** | 1 (Ref) | 25.11 (-55.17, 105.39) | 74.97 (-8.74, 158.69) | 187.99 (94.62, 281.36) | 7.56 × 10⁻⁵ | 27.23 (14.41, 40.05) | 3.44 × 10⁻⁵ |
| *^1^*Sensitivity Analysis 3: Excluding patients with prolactin levels > 4240 mIU/L (extreme outliers). A total of 985 measurements from 682 individuals were included after exclusion. 9 measurements from 2 individuals were excluded from the original dataset. Data are presented as β coefficients (95% confidence interval) for SII. PRL Quartiles Analysis: Values are median (interquartile range) for prolactin levels in mIU/L for each quartile (Quartile 1-4). The overall median (IQR) for prolactin levels is 341.30 (258.60, 459.10) mIU/L. SII: median (IQR) = 454.56 (324.86-665.53); mean ± SD = 571.82 ± 500.97. PRL Continuous Analysis: β coefficients represent the change in SII per 100 mIU/L increase in prolactin levels. Crude model: unadjusted linear mixed-effects model with random intercept for individual ID. Model 1: adjusted for age (years), sex (male or female), estimated glomerular filtration rate (eGFR, ml/min/1.73 m²), and history of hypertension, coronary heart disease, and stroke (yes or no), with random intercept for individual ID. Model 2: Model 1 + hyperlipidemia (yes or no) and hemoglobin A1c (HbA1c, %). Model 3: Model 2 + albumin (ALB, g/L) and hemoglobin (HGB, g/L). All models are linear mixed-effects models with random intercepts for individual ID to account for repeated measures. P values correspond to two-sided tests for the PRL term in each model and were reported in scientific notation when < 0.001. Abbreviations: ref, reference; SII, Systemic Immune-Inflammation Index; PRL, Prolactin; ID, Patient identification. | | | | | | | |
